# Metabolic Network Topology Reveals Transcriptional Regulatory Signatures of Type 2 Diabetes

**DOI:** 10.1371/journal.pcbi.1000729

**Published:** 2010-04-01

**Authors:** Aleksej Zelezniak, Tune H. Pers, Simão Soares, Mary Elizabeth Patti, Kiran Raosaheb Patil

**Affiliations:** 1 Center for Microbial Biotechnology, Department of Systems Biology, Technical University of Denmark, Lyngby, Denmark; 2 Center for Biological Sequence Analysis, Department of Systems Biology, Technical University of Denmark, Lyngby, Denmark; 3 Institute of Preventive Medicine, Copenhagen University Hospital, Centre for Health and Society, Copenhagen, Denmark; 4 IBB-Institute for Biotechnology and Bioengineering, Centre of Biological Engineering, Universidade do Minho, Campus de Gualtar, Braga, Portugal; 5 Research Division, Joslin Diabetes Center, Boston, Massachusetts, United States of America; King's College London, United Kingdom

## Abstract

Type 2 diabetes mellitus (T2DM) is a disorder characterized by both insulin resistance and impaired insulin secretion. Recent transcriptomics studies related to T2DM have revealed changes in expression of a large number of metabolic genes in a variety of tissues. Identification of the molecular mechanisms underlying these transcriptional changes and their impact on the cellular metabolic phenotype is a challenging task due to the complexity of transcriptional regulation and the highly interconnected nature of the metabolic network. In this study we integrate skeletal muscle gene expression datasets with human metabolic network reconstructions to identify key metabolic regulatory features of T2DM. These features include reporter metabolites—metabolites with significant collective transcriptional response in the associated enzyme-coding genes, and transcription factors with significant enrichment of binding sites in the promoter regions of these genes. In addition to metabolites from TCA cycle, oxidative phosphorylation, and lipid metabolism (known to be associated with T2DM), we identified several reporter metabolites representing novel biomarker candidates. For example, the highly connected metabolites NAD+/NADH and ATP/ADP were also identified as reporter metabolites that are potentially contributing to the widespread gene expression changes observed in T2DM. An algorithm based on the analysis of the promoter regions of the genes associated with reporter metabolites revealed a transcription factor regulatory network connecting several parts of metabolism. The identified transcription factors include members of the CREB, NRF1 and PPAR family, among others, and represent regulatory targets for further experimental analysis. Overall, our results provide a holistic picture of key metabolic and regulatory nodes potentially involved in the pathogenesis of T2DM.

## Author Summary

Type 2 diabetes mellitus is a complex metabolic disease recognized as one of the main threats to human health in the 21^st^ century. Recent studies of gene expression levels in human tissue samples have indicated that multiple metabolic pathways are dysregulated in diabetes and in individuals at risk for diabetes; which of these are primary, or central to disease pathogenesis, remains a key question. Cellular metabolic networks are highly interconnected and often tightly regulated; any perturbations at a single node can thus rapidly diffuse to the rest of the network. Such complexity presents a considerable challenge in pinpointing key molecular mechanisms and biomarkers associated with insulin resistance and type 2 diabetes. In this study, we address this problem by using a methodology that integrates gene expression data with the human cellular metabolic network. We demonstrate our approach by analyzing gene expression patterns in skeletal muscle. The analysis identified transcription factors and metabolites that represent potential targets for therapeutic agents and future clinical diagnostics for type 2 diabetes and impaired glucose metabolism. In a broader perspective, the study provides a framework for analysis of gene expression datasets from complex diseases in the context of changes in cellular metabolism.

## Introduction

Type 2 diabetes mellitus (T2DM) is emerging as one of the main threats to human health in the 21^st^ century with an estimated 300 million individuals with T2DM by the year 2025 [1],[2]. T2DM is characterized by both insulin resistance (as manifested by reduced insulin-stimulated glucose uptake in skeletal muscle and adipose tissue and inappropriately high hepatic glucose output [3],[4]) and reduced insulin secretion by pancreatic β-cells [3],[5]. Although the specific molecular pathophysiology remains unclear, many risk factors have been identified for T2DM, including family history of diabetes and prominent environmental factors such as alterations in early life development, excessive food intake, obesity, decreased physical activity and aging [2],[3],[5]. At the cellular level, multiple regulatory mechanisms and metabolic pathways may contribute to the pathogenesis of insulin resistance, potentially mediated by alterations in insulin signaling [6], mitochondrial oxidative metabolism and ATP production [7]–[9], fatty acid oxidation [10], or proinflammatory signaling [11]. Similarly, alterations in β-cell development and metabolism [5] may contribute to decreased insulin secretion.

Available human tissue transcriptome data related to T2DM [12],[13] provide an opportunity for identification of novel molecular mechanisms underlying the metabolic phenotype of T2DM. This task is challenging due to the need to account for the inherent high connectivity of bio-molecular interaction networks. We have utilized a network-centered methodology to link diabetes-related alterations in gene expression to metabolic hot spots and transcription factors potentially responsible for gene expression changes.

### Rationale and methodology

Metabolic phenotypes at a cellular level are essentially characterized by concentrations of metabolites and fluxes through the reactions that make up the metabolic network. Fluxes, in turn, are dependent on metabolite levels, enzyme activities, abundance of effectors and possibly other variables. Measurement of fluxes and metabolite concentrations at the entire metabolic network-scale is, however, a difficult task in humans due to a variety of technological and experimental limitations. By contrast, methods for measurement of expression of genes encoding metabolic enzymes are relatively well-established. Thus, the primary goal of this study is to use informatics approaches to integrate available gene expression data with metabolic networks, in order to predict metabolic phenotypes of skeletal muscle linked to the pathogenesis of type 2 diabetes. Such an approach will help not only to gain insight into the organization of transcriptional regulation in human tissues, but also provide guidance for improved design of experimental strategies for obtaining metabolite and flux data, which can be further integrated into metabolic models.

**Figure 1:**
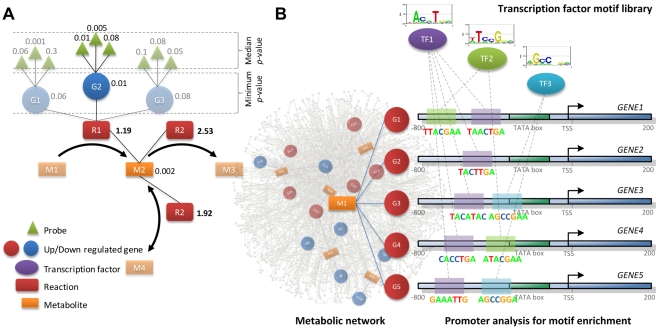
Schematic overview of the methodology used for the identification of reporter metabolites and associated putative regulatory sequence motifs. A) Scoring system for identification of reporter metabolites. Each metabolite is scored based on the scores of the associated enzyme-catalyzed reactions. Each enzyme, in turn, is assigned a score based on median of the p-values of the probes representing the corresponding gene. In case of a reaction catalyzed by an enzyme complex or a set of isozymes, minimum of the p-values of the corresponding enzymes is chosen. Numbers in bold are Z-scores for each reaction, the rest of the numbers represent p-values (significance of differential expression). B) Identification of transcription factor binding motifs. For a reporter metabolite, a set of up/down regulated neighbor (enzyme-coding) genes is selected. Promoter regions, upstream of transcription start site (TSS) of each of the selected genes are assessed for the enrichment of known transcription factor (TF) binding sequence motifs.

To achieve these goals, we applied an extension of the algorithm described in [14] (for various applications of this algorithm see [14]–[18]), which enables identification of so-called reporter metabolites, or metabolic hot spots around which transcriptional regulation is centered (Figure 1A). This analysis is based on the assumption that under most conditions of physiological interest, fluxes through enzymes connected to a metabolite are coordinated in order to maintain physiological homeostasis, or to eventually reach a new (pseudo-) steady state. Moreover, transcriptional regulation of expression of genes encoding critical enzymes in metabolic flux pathways facilitates concordance with the metabolic demands of the cell and corresponding stoichiometric and thermodynamic constraints on fluxes. For this analysis, we used two recently published human metabolic network models: i) *Homo sapiens* Recon1 [19], and ii) Edinburgh Human Metabolic Network (EHMN) [20].

We further hypothesized that the observed coordinated changes around reporter metabolites can be, at least in some cases, attributed to common transcriptional regulatory mechanisms. Specifically, we hypothesize that the neighbor enzymes of reporter metabolites may share one or more transcription factor binding sites in the promoter regions of the corresponding genes. In order to identify such potential regulatory players, we tested promoter sequences of the genes associated with the reporter metabolites for enrichment of known transcription factor binding motifs (Figure 1B). Transcription factors identified in this fashion provide clues to the regulatory mechanisms that lead to observed gene expression changes in the metabolic network.

Since our goal is to identify reporter metabolites and transcription factors potentially involved in diabetes pathogenesis and progression, we analyzed two independent studies of skeletal muscle transcriptomics in individuals with established type 2 diabetes or insulin resistance [8],[9] (Text S1). In the first study [8], biopsies were obtained following insulin stimulation from a cohort of 43 Swedish men of Caucasian ethnicity with a spectrum of glucose tolerance, including 17 with normal glucose tolerance (NGT), 8 with impaired glucose tolerance (IGT), and 18 with established T2DM. The second dataset [9] was derived from a cohort of 15 subjects of Mexican American ethnicity, in whom muscle biopsies were performed in the fasting state. Importantly, this cohort included individuals with not only established diabetes (5 subjects, T2DM), but also individuals with completely normal glucose tolerance but a spectrum of insulin resistance; normal glucose tolerant subjects were subdivided by family history-linked diabetes risk (4 family history positive, more insulin resistant subjects, FH+; and 6 family history negative, more insulin sensitive subjects, FH−). With this approach, the individual contributions of isolated insulin resistance and diabetes risk (in the setting of normoglycemia, FH+), mild elevations in postprandial glucose (IGT), and established diabetes can be individually assessed. Moreover, the possible contribution of family history, potentially mediated by genetics or shared environment, can be assessed. Thus, we predict that analysis of the common patterns resulting from the two datasets will identify regulatory signatures potentially independent of study cohort and design variation but common to the pathophysiology of insulin resistance and diabetes.

## Results

In present study, we performed reporter metabolite analysis based on pair-wise comparisons within each dataset; differential expression and its significance were assessed with robust multi-array average (RMA) and empirical Bayes testing. Significance of differential expression for each gene was used as a scoring metric (Materials and Methods). The results are summarized as metabolic signatures (reporter metabolites) and regulatory signatures (transcription factors) for T2DM.

### Metabolic signatures of T2DM

#### Swedish male dataset

Reporter metabolite analysis for three pair-wise comparisons, *viz.*, T2DM *vs* NGT, T2DM *vs* IGT, and IGT *vs* NGT, revealed significant reporter metabolites (p-value≤0.05) participating in lipid metabolism, TCA cycle, oxidative phosphorylation (OXPHOS) and glycolysis (Table 1, Table 2, Table S1 and Table S2). Among reporter metabolites identified for the T2DM *vs* NGT comparison were lipid species 1,2-diacyl-sn-glycerol (DAG), acetoacetyl-CoA, and the sphingolipid sphinganine. These are interesting, as prior studies [3], [21]–[23] have demonstrated that the related lipid molecules diacylglycerols (DAG), long-chain fatty acyl CoAs, and ceramides correlate positively with triglyceride content and inversely with insulin sensitivity [5] and have been shown to induce insulin resistance [3]. Furthermore, given that saturated fatty acids appear to play a particularly important pathogenic role in insulin resistance [24], it is interesting that several metabolites of saturated fatty acids (such as hexanoyl-CoA, palmitoyl-CoA, tetradecanoyl-CoA, lauroyl-CoA, decanoyl-CoA and butanoyl-CoA) were found as reporter metabolites with mostly up-regulated neighboring genes in the IGT *vs* NGT comparison (Table 1 and S2), and thus may serve as potential markers of insulin resistance and IGT.

**Table 1:** Reporter metabolites for Swedish male dataset.

Reporter Metabolite	P-values		Enzyme neighbors (Up-regulated∶Down-regulated)		
	T2DM/NGT	IGT/NGT	T2DM/NGT	IGT/NGT	
*Citrate*	**0.047**	0.646	1∶0	1∶0	TCA cycle
Succinyl-CoA	**0.013**	0.285	2∶3	2∶3	
2-Hydroxyglutarate*	**0.002**	**0.023**	0∶1	0∶1	
*2-Oxoglutarate* *	**0.049**	**0.047**	8∶11	8∶11	
Ferrocytochrome C; Ferricytochrome C	**0.006**	**0.032**	1∶2	0∶3	Oxidative phosphorylation
Ubiquinone-10	**0.017**	0.769	0∶5	1∶4	
Ubiquinol-10	**0.022**	0.484	0∶4	1∶3	
Phosphoenolpyruvate*	0.196	**0.037**	1∶3	1∶3	Glycolysis
D-Glyceraldehyde*	0.083	**0.017**	2∶1	3∶0	
*D-Alanine*	**0.016**	0.330	0∶3	0∶3	Amino acid metabolism
*L-Alanine*	**0.047**	0.319	3∶7	3∶7	
3-Methylglutaconyl-CoA†	**0.038**	0.816	0∶2	1∶1	
*L-Leucine* *	**0.047**	0.109	1∶3	1∶3	
*1,2-Diacyl-sn-glycerol (DAG)* *	**0.022**	**0.049**	2∶5	2∶5	Lipid metabolism
1D-myo-Inositol 1,4-bisphosphate†	0.060	0.151	0∶3	2∶1	
3-Dehydrosphinganine*	0.232	**0.035**	1∶1	2∶0	
Acetoacetyl-CoA*	**0.009**	0.462	1∶4	2∶3	
Butanoyl-CoA†	0.365	**0.038**	0∶2	1∶1	
*Decanoyl-CoA; Lauroyl-CoA* *	0.268	**0.033**	1∶2	2∶1	
Fatty acid*	**0.021**	0.756	3∶4	3∶4	
Lophenol* §	**0.007**	0.749	0∶1	0∶1	
*Palmitoleoyl-CoA* *	0.238	**0.019**	1∶3	2∶2	
*Palmitoyl-CoA* *	0.179	**0.014**	3∶4	6∶1	
Phosphatidyl glycerol phosphate	**0.047**	0.316	0∶1	0∶1	
Phosphatidylinositol 4,5-bisphosphate	0.097	**0.001**	1∶5	2∶4	
Propanoyl-CoA*	0.259	**0.016**	2∶5	2∶5	
Prostaglandin E2	**0.036**	**0.032**	0∶3	1∶2	
Sphinganine*	**0.038**	0.283	1∶3	2∶2	
(Gal)3 (GalNAc)1 (Glc)1 (Cer)1*	**0.023**	**0.034**	1∶2	1∶2	Other
AMP†	**0.041**	0.218	7∶17	6∶17	
ATP†	**0.003**	**0.010**	28∶60	27∶60	
cAMP†	**0.033**	**0.049**	2∶0	2∶0	
CDPcholine	**0.020**	0.122	0∶2	0∶2	
Choline phosphate	**0.030**	0.573	0∶2	1∶1	
NAD+*	0.333	**0.020**	29∶34	34∶34	
*Phosphocreatine*	**0.025**	0.176	0∶1	1∶0	
Trichloroethanol*	**0.020**	**0.038**	1∶2	3∶0	

*Reporter metabolites identified using EHMN metabolic network.

**†:** Reporter metabolites identified in both networks.

**§:** Plant metabolite, likely to be present in the EHMN due to incorrect annotation.

Reporter metabolites with p≤0.05 in at least one of the comparisons showed in bold. Columns with enzyme neighbors show the number of up- and down-regulated enzyme neighbors in the first condition (e.g. T2DM/NGT up- and down-regulated in T2DM comparing with NGT) for each of comparisons. Reporter metabolites without marks were identified using Recon1 metabolic network. Metabolites written in italics are known to be directly/indirectly related to T2DM, see main text and Table S8.

**Table 2:** Reporter metabolites for Mexican-American dataset.

Reporter metabolite	P-values		Enzyme neighbors (Up-regulated∶Down-regulated)		
	T2DM/FH−	FH+/FH−	T2DM/FH−	FH+/FH−	
*2-Oxoglutarate*	**0.001**	**0.001**	2∶7	2∶7	TCA cycle
*L-Malate*	0.098	**0.029**	1∶4	2∶3	
Succinyl-CoA†	**0.011**	**0.009**	0∶5	0∶5	
Ferrocytochrome C;Ferricytochrome C	**0.008**	**0.007**	0∶3	0∶3	Oxidative phosphorylation
*Fumarate*	**0.019**	**0.025**	0∶2	0∶2	
Ubiquinone-10†;Ubiquinol-10†	**0.040**	**0.021**	1∶3	1∶3	
2,3-Disphospho-D-glycerate†	**0.021**	**0.004**	0∶1	0∶1	Glycolysis
2-Phospho-D-glycerate*	**0.038**	**0.006**	0∶2	1∶1	
beta-D-Fructose*	**0.049**	**0.038**	0∶2	0∶2	
D-Fructose 2,6-bisphosphate	**0.037**	0.136	0∶2	0∶1	
D-Fructose 6-phosphate	**0.013**	0.119	4∶6	3∶7	
D-Glucose*	**0.037**	0.066	0∶7	1∶5	
D-Glucose 6-phosphate	**0.009**	**0.014**	1∶3	1∶3	
D-Glycerate 2-phosphate	**0.026**	**0.003**	0∶2	1∶1	
*L-Lactate*	**0.048**	0.067	1∶2	1∶2	
Phosphoenolpyruvate	0.079	**0.048**	2∶2	3∶1	
*Pyruvate*	**0.042**	0.202	1∶6	1∶6	
2-Oxoadipate*	**0.002**	**0.004**	0∶1	0∶1	Amino acid metabolism
*beta-Alanine*	**0.031**	**0.027**	1∶1	1∶1	
*L-Glutamate* †	**0.025**	**0.009**	1∶1	1∶1	
(R)-2-Methyl-3-oxopropanoyl-CoA*	**0.043**	0.118	0∶2	0∶1	Lipid metabolism
*1,2-Diacyl-sn-glycerol (DAG)* *	**0.036**	0.117	3∶2	5∶1	
1D-myo-Inositol 1,4-bisphosphate	**0.025**	0.054	1∶2	1∶2	
3-cis-Dodecenoyl-CoA*	**0.009**	**0.039**	0∶3	0∶3	
Acylglycerol*; 2-Acylglycerol*	**0.035**	**0.018**	1∶1	1∶1	
Glutaryl-CoA†	**0.007**	**0.015**	0∶2	0∶2	
*Glycerol*	**0.020**	**0.001**	1∶1	1∶1	
*Glycerol 3-phosphate*	0.051	**0.005**	2∶1	2∶1	
Lipoamide*	**0.014**	**0.006**	0∶5	0∶5	
Phosphatidylinositol	**0.017**	0.128	1∶5	1∶5	
trans-3-decenoyl-CoA*	**0.026**	0.076	0∶2	0∶2	
ADP	**0.047**	0.174	16∶31	20∶27	Other
CO_2_	**0.041**	**0.004**	1∶11	3∶9	
Coenzyme A†	**0.007**	**0.014**	4∶8	3 10	
*Creatine;Phosphocreatine* †	**0.032**	**0.048**	0∶1	0∶1	
NAD+†; NADH†	**0.003**	0.095	3∶17	17∶4	
Trichloroethanol*	**0.021**	**0.006**	2∶1	3∶0	

*: Reporter metabolites identified using EHMN metabolic network.

**†:** Reporter metabolites identified in both networks.

Reporter metabolites with p≤0.05 in at least one of the comparisons showed in bold. Columns with enzyme neighbors show the number of up- and down-regulated enzyme neighbors in the first condition (e.g. T2DM/FH− up- and down-regulated in T2DM comparing with FH−). Reporter metabolites without marks were identified using Recon1 metabolic network. Metabolites written in italics are known to be directly/indirectly related to T2DM, see main text and Table S8.

TCA cycle metabolites citrate and 2-oxoglutarate, with down-regulated neighboring genes, were also uncovered as reporter metabolites in the T2DM *vs* NGT comparison (Table 1, S1 and S2). These results are concordant with a study of human urine metabolome profiles from patients with T2DM [25], in which levels of citrate and 2-oxoglutarate were lower in T2DM compared to healthy controls [26]. Among other mitochondrial metabolites, reduced and oxidized forms of cytochrome c and ubiquinol were identified as reporter metabolites (T2DM *vs* NGT, Table S1) with down-regulated expression of the associated genes.

Impaired glucose tolerance typically reflects an important transition between normoglycemia and overt diabetes, reporter metabolites which are identified in both IGT *vs* NGT and T2DM *vs* NGT, but not significantly different in the T2DM *vs* IGT comparison (e.g. phosphatidylethanolamine, 2-hydroxyglutarate, 2-oxoglutarate, 3′,5′-cyclic AMP, ATP, Table S1 and S2) may be considered novel biomarkers of early-stage glucose intolerance.

#### Mexican-American dataset

We similarly performed reporter metabolite analysis using both Recon1 and EHMN metabolic models in the Mexican-American dataset. This analysis revealed significant transcriptional regulation in metabolite nodes in TCA cycle, oxidative phosphorylation, and lipid metabolism, for both T2DM *vs* FH− and FH+ *vs* FH− comparisons (Table 2). Similar to the Swedish Caucasian dataset, metabolites involved in oxidative phosphorylation (e.g. ferrocytochrome c, H+, and fumarate) were among the top-ranking reporter metabolites, identified in both the T2DM *vs* FH− and FH+ *vs* FH− comparisons (Table 2, Table S3). Interestingly, urinary levels of fumarate, an important link between the TCA cycle and oxidative phosphorylation, were recently found to be decreased in T2DM patients [25].

Analysis using the EHMN model revealed TCA cycle-related metabolites, including 3-carboxy-1-hydroxypropyl-ThPP, aconitate, succinyl-CoA, malate and fumarate, as significant reporter metabolites (p-value≤0.05), with mostly down-regulated expression of the genes encoding their neighboring enzymes. Ubiquinol was found as reporter metabolite representative of electron transfer chain. Several molecules within β-oxidation pathways, such as 3-cis-dodecenoyl-CoA, glutaryl-CoA, trans-3-decenoyl-CoA, 3-methylbutanoyl-CoA and 3-methylcrotonyl-CoA, as well as in amino acid (leucine, lysine) metabolism were also identified as reporters (Table 2, Table S4). Moreover, glutamate, glycerol derivatives, phosphocreatine, a number of hormone derivatives and many others (Table S3 and S4) were found as significant reporter metabolites in the T2DM *vs* FH− comparison.

#### Overlapping reporter metabolites between two study populations

**Figure 2:**
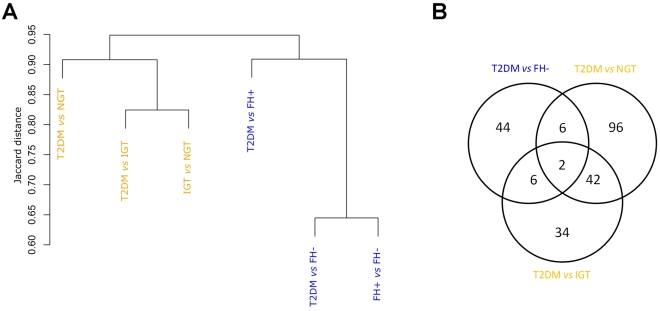
Hierarchical clustering of pair-wise comparisons within the Swedish male and Mexican-American datasets based on the overlapping reporter metabolites (Recon1 model). Comparisons are colored according to the dataset; blue – Mexican-American; orange – Swedish male dataset. A) Dendrogram of reporter metabolites identified in each of the comparisons based on Jaccard distance. B) Venn diagram showing the overlap of the reporter metabolites identified in the different comparisons.

In order to determine the extent of overlap between the two study populations, we performed a cluster analysis of the pair-wise comparisons within the Swedish and Mexican-American datasets (Figure 2). Jaccard distance metric between two pair-wise comparisons (e.g. T2DM *vs* FH− and FH+ *vs* FH−) was calculated based on the overlap of reporter metabolites between the two comparisons. Jaccard distance provides a measure of dissimilarity between two sets of reporter metabolites, and is quantified as the fraction of non-overlapping reporter metabolites between the two sets. While similar clustering patterns were observed (Figure 2A and Figure S1A) independent of the use of either EHMN or Recon1 metabolic model, Swedish and Mexican-American studies clustered separately, perhaps related to differences in study population, study design (e.g. fasting studies in Mexican-Americans, insulin-stimulated studies in Swedish) or differences in microarrays used (thus differing in the coverage of metabolic enzymes). We observed substantial overlap between the T2DM *vs* FH− and FH+ *vs* FH− comparisons, suggesting that insulin resistance patterns could contribute to these findings.

We next examined the overlap of reporter metabolites between the two case studies (Figure 2B, Figure S1B, Table S5 and Table S6). Owing to differences in the metabolite-gene connectivity between EHMN and Recon1, the number of overlapping reporter metabolites is generally higher for the EHMN analysis. To a large extent, this difference is due to the groups of metabolites in EHMN that share the same gene neighbors (whether two metabolites share the same gene neighbors depends not only on the network used, i.e. number of distinct biochemical reactions associated with a particular enzyme, but also on the coverage of genes on the particular microarray chip used). In addition to many other metabolites, phosphocreatine appeared as a significant reporter in both case studies, *viz.*, for T2DM *vs* NGT and T2DM *vs* FH− comparisons. Phosphocreatine is an important energy reservoir metabolite in skeletal muscle, and defects in recovery of phosphocreatine have been identified *in vivo* in humans with insulin resistance [27] and diabetes [28]. Interestingly, low levels of urinary creatine have also been found in patients with T2DM [25].

### Regulatory signatures of T2DM

In order to link the identified reporter metabolites to regulatory pathways controlling gene expression, we hypothesized that enzymes associated with reporter metabolites would be regulated by common transcription factors. As potential candidates subjected to such regulation, we selected all reporter metabolites with at least 5 up- or down-regulated neighboring genes (Materials and Methods). Up- and down-regulated gene sets were then analyzed separately in order to assess whether their promoter regions were enriched for known transcription factor binding sequence motifs. P-values for enrichment were estimated by using a hypergeometric test, which compared the proportion of promoters from a given gene set containing a particular motif with the frequency of occurrence of that motif in promoter regions of all other metabolic genes. Correction for multiple-testing was done by using q-value [29] and motifs with q-value≤0.05 were considered as significantly enriched.

**Figure 3:**
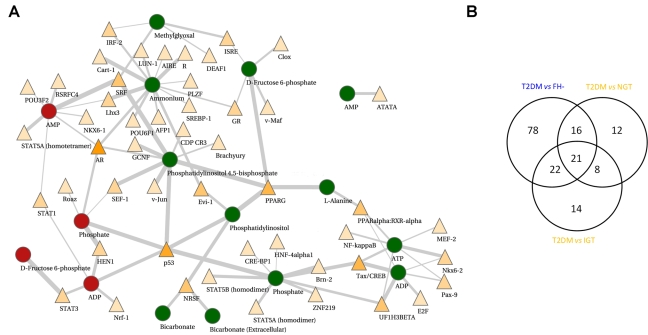
Summary of the main results from the motif enrichment analysis. A) Motif enrichment analysis for the genes associated with reporter metabolites from the T2DM *vs* NGT comparison. Reporter metabolites with up-regulated neighboring gene set are shown as red circles, whereas reporter metabolites with down-regulated neighboring gene set are represented as green circles. Transcription factor binding motifs (shown as triangles) are colored according to the number of enzyme sets in which they are enriched, ranging from light yellow (enriched in few sets) to orange (enriched in as many as 6 sets). Edges are scaled according to q-values signifying the confidence of the motif enrichment. B) Venn diagram showing the overlap of transcription factor binding motifs across the comparisons of T2DM with non-T2DM cases. Comparisons are colored according to the dataset; blue – Mexican-American; orange – Swedish male dataset.

In accord with our hypothesis, several transcription factor binding sites were overrepresented in the promoter regions of the enzymes associated with reporter metabolites. A summary of the main results from this analysis is illustrated in Figure 3A. Many transcription factors were found to be common across the two case studies (Figure 3B), *albeit* in connection with different reporter metabolites. PPAR family motifs (PPARγ and PPARα:RXRα) were enriched in seven downregulated enzyme sets including ATP. Tax/CREB motifs were enriched in promoters of downregulated enzymes associated with ATP, ADP and phosphate. Additional down-regulated neighbors of ATP were enriched for the binding sites of NF-κB, MEF-2, UF1-H3β, Pax-9 and NKX6.2, while the NRF-1 motif was enriched in the set of up-regulated enzymes neighboring ADP. Another potential regulatory signature was identified around the down-regulated neighbors of phosphatidylinositol and phosphatidylinositol 4,5-bisphospate (important phospholipids which participate in insulin and other signaling reactions), which were significantly enriched for binding sites of p53, PPARγ, SRF, SEF-1, v-Jun, GCNF, AR and many others (Table S7). These and other highly connected reporter metabolites in the metabolite-TF network (Figure 3A) demonstrate the concept that associated metabolic pathways can be transcriptionally regulated in multiple ways in response to environmental stimuli or metabolic perturbation.

## Discussion

Maintenance of whole-body glucose metabolism is reliant on a delicately balanced dynamic interaction between tissue sensitivity to insulin (including muscle, adipose and liver) and insulin secretion [5],[30]. Unfortunately, the molecular mechanisms responsible for diabetes risk remain unknown. A key metabolic phenotype associated with insulin resistance in humans is inappropriate lipid accumulation in tissues outside of adipose tissue, suggesting defects in fatty acid uptake, synthesis, and/or oxidation. With lipid excess and/or impaired oxidation, as observed in obesity and/or inactivity, flux of long-chain acyl CoAs (LC-CoA) may be redirected into cytosolic lipid species such as diacylglycerols (DAG), triacylglycerols (TG) and ceramides (derivatives of sphingosine and fatty acid metabolism) [5] that are correlated with reductions in insulin signaling and insulin resistance [3], [21]–[23],[31]. Whether alterations in mitochondrial oxidative function in humans with insulin resistance and diabetes contribute to, or are a consequence of these defects, remains unclear [32].

**Figure 4:**
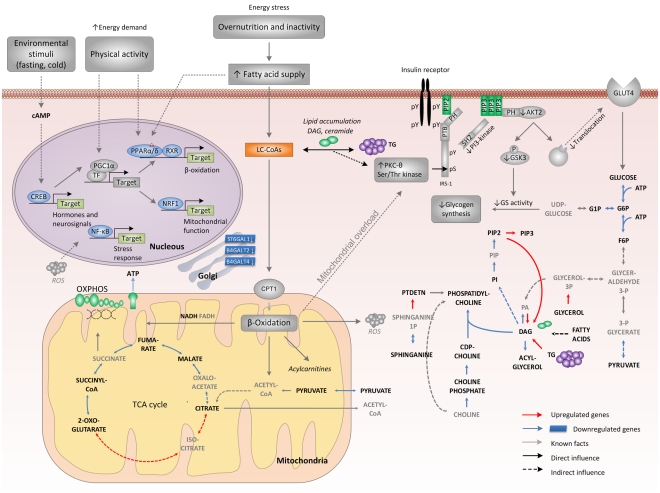
Metabolic and regulatory signatures of type 2 diabetes. Key metabolic and regulatory pathways associated with reporter metabolites identified in this study (T2DM *vs* NGT and T2DM *vs* FH− comparisons) are shown. Metabolites in bold black font are reporter metabolites. Grey shapes and arrows represent facts/hypotheses from previous studies and are not directly based on the results from the present study. Broken lines imply indirect effect while full lines denote direct effect. Chronic overfeeding and physical inactivity increase the influx of fatty acid, which promotes β-oxidation through the activation of PPARα/δ-mediated genes, without coordinated increase in TCA cycle flux. Reporter analysis supports this idea by showing the decreased activity in TCA cycle enzymes associated with reporter metabolites. Eventually, this leads to mitochondrial accumulation of metabolic by-products of incomplete β-oxidation (acylcarnitines ROS). These stresses might lead to mitochondrial overload which together with intracellular lipid-signaling (such as DAG) molecules might trigger serine a serine/threonine (Ser/Thr) kinase (Ser/Thr) cascade initiated by nPKCs. As a result, Ser/Thr phosphorylation of insulin receptor substrate 1 (IRS-1) sites is induced, thereby inhibiting IRS-1 tyrosine phosphorylation and activation of PI 3-kinase, resulting in impeded GLUT4 translocation, reduced glucose transpor, and decreased glycogen synthesis. Increased physical activity/fasting activates PGC1α and CREB (a potent inducer of PGC-1α). These actions combat lipid stress by increasing TCA cycle flux and by coupling ligand-induced PPARα/δ activity with PGC1α-mediated remodeling of downstream metabolic pathways such as respiration and β-oxidation. CDP-choline, cytidine diphosphate choline; DAG, diacylglycerol; G1P, glucose 1-phosphate; G6P, glucose 6-phosphate; GLUT4, glucose transporter-4; GSK3, glycogen synthase kinase-3; IRE1, inositol requiring kinase-1; LC-CoAs, long-chain acyl CoAs; nPKCs, novel protein kinase Cs; PA, phosphatidate; PGC1α, PPARγ co-activator-1α; PH, pleckstrin homology domain;PI, phospatidylinositol; PIP, phospatidylinositol 4-phospate; PIP2, phosphatidylinositol 4,5-bisphospate, PIP3, phospatidylinositol 3,4,5-trisphospate; PI 3-kinase, phosphoinositol 3-kinase; PPARγ, peroxisome proliferator-activated receptor-γ; PTB, phosphotyrosine binding domain; ROS, reactive oxygen species; RXR, retinoid X receptor; SH2, src homology domain; TCA, tricarboxylic acid cycle; TF, transcription factor; CPT1, carnitine palmitoyltransferase-1; PTDETN, phosphatidylethanolamine.

Recognizing these important gaps in our knowledge of diabetes pathophysiology, we have integrated transcriptomic data with metabolic networks to systematically identify, in an unbiased fashion, regulatory hot spots (reporter metabolites and associated transcription factors) associated with insulin resistance and T2DM. Our reporter metabolite results provide evidence for transcriptional dysregulation of multiple metabolic pathways in skeletal muscle. Interestingly, many of the reporter metabolites identified in our analysis have been appreciated in prior experimental studies in animal models (metabolites with italic font in Tables 1, 2 and S8). A bird's-eye view of selected metabolic and regulatory nodes identified in our study is depicted in Figure 4.

### Key metabolic regulatory nodes in T2DM pathogenesis

#### Lipid metabolism

In conditions of overnutrition and physical inactivity, availability of cellular fatty acids stimulate ligand–dependent PPARα/δ transcription factors which, in turn, induce transcription of genes responsible for β-oxidation [33],[34]. Metabolic byproducts of incomplete β-oxidation, such as acylcarnitines and reactive oxygen species, may accumulate in mitochondria and contribute to insulin resistance [5]. Interestingly, our analysis identified enrichment of PPAR family transcription factor binding motifs in T2DM as compared with insulin sensitive subjects, in both the Swedish and Mexican-American datasets (T2DM *vs* NGT and T2DM *vs* FH−, respectively). Moreover, reporter analysis revealed lipid metabolites (Table S1), known to be natural ligands of PPARγ (prostaglandins) [34].

Another reporter metabolite identified in our analysis is diacylglycerol (DAG), a lipid signaling molecule known to inversely correlate with insulin sensitivity [3], [21]–[23],[31]. Our results suggest that perturbations in DAG levels may be accompanied by changes in the adjacent CDP-Choline branch of the Kennedy pathway of phospholipid metabolism (Figure 4). Thus, DAG could potentially affect insulin sensitivity *via* activation of serine/threonine kinases or alterations in phospholipid membrane composition, both of which could lead to defects in insulin signaling, reduced insulin-stimulated glucose uptake, and glycogen synthesis – key metabolic features of diabetes [5] (Figure 4). Together, identification of these lipid-linked regulatory motifs and reporter metabolites known to be involved in type 2 diabetes pathogenesis provides further support for the validity of our approach.

#### Central carbon metabolism

Using our approach we found several reporter metabolites from the TCA cycle (citrate, 2-oxoglutarate, succinyl-CoA, fumarate and malate) (Figure 4). The down-regulated genes associated with these metabolites support the idea that TCA cycle and/or oxidative phosphorylation flux is reduced in diabetes [9]. It is also interesting that ATP is one of the reporter metabolites, as the majority of cellular ATP is generated *via* respiration. Moreover, significant enrichment of binding motif for NF-κβ in the upregulated ATP neighbors is consistent with the potential role of this transcription factor in mediating oxidative stress responses triggered by by-products of incomplete β-oxidation [35]. Another interesting finding is the enrichment of CREB family and NRF-1 motifs in enzymes associated with ATP and ADP. These results corroborate the role of CREB as an indirect regulator of nuclear-encoded oxidative phosphorylation genes *via* PGC1-α and other regulators linked to nuclear-encoded mitochondrial genes (Figure 4) [9],[36],[37].

The appearance of highly connected metabolites, such as ATP and NADH, among top-ranking reporter metabolites provides a possible link to the observed network-wide transcriptional changes in IGT and T2DM. Cellular levels of these co-factors are usually constrained within relatively narrow ranges to maintain thermodynamic stability. Oxidative phsophorylation, which is connected to TCA cycle flux *via* succinate and fumarate, accounts for most of the ATP (and NADH) turnover in a respiring cell. Our results suggest reduction in the activity of both TCA cycle and oxidative phosphorylation, in agreement with recent NMR data demonstrating that mitochondrial ATP synthesis is reduced in humans with insulin resistance [38]–[40]. Another major source of ATP and NADH production in the cell is glycolysis. Reporter metabolites representative of glycolysis (glucose, glucose-6-phosphate, glucose-1-phosphate and pyruvate) also exhibited concordant down-regulation of the neighboring genes.

The concordance between the changes in gene expression levels for glycolysis, TCA cycle and oxidative phosphorylation in IGT and T2DM suggests that transcriptional regulatory mechanisms may be a response to altered levels of ATP/NADH. Such response may achieve two purposes: (1) regulation of metabolism on global scale, as these co-factors are critical components of many metabolic pathways, and (2) regulation of NADH levels may help in reducing excessive (and potentially deleterious) oxidative stress resulting from sustained oxidation of excessive nutrients [41]. Although the way such regulatory control is mechanistically linked to the corresponding metabolites cannot be deduced from the gene expression data alone, there are several examples where metabolite co-factors are directly involved in regulating gene expression, e.g. NADH(/+) dependent regulation of genes in gram-positive bacteria [42], yeast [43]–[45] and human [46],[47]. NAD+ dependent changes in gene expression levels could also be mediated by the action of PGC-1α and SIRT1 complex, which have important roles in regulation of glucose homeostasis [48]. Additional regulatory links, between glycolytic flux, energy metabolism, TCA cycle flux and fatty acid metabolism are also known in other eukaryotic systems such as baker's yeast [49]–[51]. Furthermore, several of the enzymes from central carbon metabolism may be regulated to a large extent at the post-transcriptional level [52],[53]. Parallels of such regulatory circuits in human cells may be discovered in the future with the here-identified transcription factors (Table S7) as one of the starting points.

#### Other pathways

Metabolites involved in protein and lipid glycosylation were found as reporters and characterized by down-regulation of neighboring enzymes (Table S2). Alterations in glycosylation may ultimately cause misfolding of several proteins, a feature previously associated with over-nutrition in hepatocytes [5]. Another reporter metabolite, shared by T2DM *vs* NGT and T2DM *vs* FH− comparison, is trichloroethanol, a metabolite in the cytochrome P450-mediated pathway derived from trichlorethene [54]. Although tricholoethanol or tricholoethene is not an endogenous metabolite in human tissues, it appears that the expression of the cytochrome P450 is altered in T2DM. Interestingly, experimental evidence shows that mouse exposure to trichlorethene leads to PPARα activation and the reprogramming of gene expression, resulting in induction of enzymes mediating β- and ω-oxidation of fatty acids, and increased expression of genes involved in lipid metabolism [55], a pattern similar to the T2DM metabolic phenotype [3].

### Reporter metabolites and macroscopic physiological parameters

**Figure 5:**
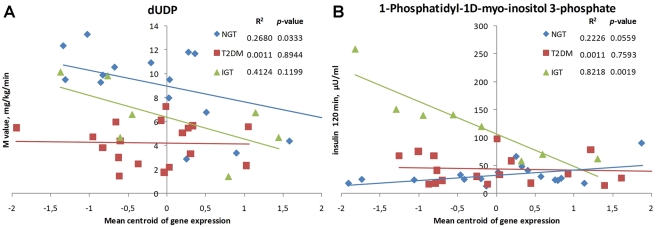
Correlation of glucose uptake and insulin level with mean centroid expression levels of reporter metabolite neighbor genes (Swedish male dataset). M value – whole-body glucose uptake during the hyperinsulinemic euglycemic clamp, Insulin 120 min – insulin levels achieved at the two hour time point of oral glucose tolerance test.

The identification of reporter metabolites from glycolysis and energy-generation pathways suggests that there may be regulation of certain physiological parameters, such as glucose uptake, at the transcriptional level of the corresponding metabolic pathways. To investigate the extent of such possible regulation, we calculated Pearson correlation coefficients between insulin sensitivity (as measured by either whole-body glucose uptake during the hyperinsulinemic euglycemic clamp or insulin levels achieved during the OGTT) and mean centroid expression levels of genes surrounding reporter metabolites (Swedish dataset) (Materials and methods). A significant linear correlation with whole-body glucose uptake was observed for several reporter metabolites. In most cases, the correlation was significant only for one of the conditions (NGT, IGT or T2DM). For example, significant correlation of transcriptional regulation around dUDP with glucose uptake was found only for NGT samples (Figure 5A). It appears that this potential connection is de-linked under IGT and T2DM conditions. Another example is 1-Phosphatidyl-1D-myo-inositol 3-phosphate (Figure 5B), where significant correlation is observed with insulin level only for IGT. Further investigation of the causal mechanisms behind these observed correlation patterns may help in elucidating the regulatory role of the reporter metabolites in diabetes pathogenesis.

### Potential biomarkers and pharmacological targets

A key scientific and clinical challenge is to identify molecular markers of diabetes risk, not only to better understand disease pathophysiology, but also to develop novel therapies for prevention and treatment of established diabetes. In this context, it is interesting that our analysis identified both PPARγ and its potential lipid ligands as regulatory molecules, since PPARγ ligand thiazolidinediones are currently employed as effective therapy for diabetes. We hypothesize that some transcriptional pathways identified in the current analysis, including CREB, NRF-1 and SRF, may be additional novel molecular mediators of the transcriptomic phenotype associated with insulin resistance, and thus potential targets for future intervention strategies. Of course, the potential roles of these pathways will require additional testing in cultured cells and animal models, where their impact on metabolic flux and insulin sensitivity can be fully assessed.

Similarly, reporter metabolites identified in our analysis represent molecules likely to be involved in human skeletal muscle insulin resistance phenoytpes and also novel candidate biomarkers of insulin resistance and diabetes risk. In support of this hypothesis, several of the identified metabolites have known physiological roles in T2DM (Table S8 and Discussion above). Additional molecules have been analyzed either in rodents and/or in other tissues (Table S8) and thus, their appearance as reporter metabolites also strongly implicates their involvement in insulin resistance in human skeletal muscle. Some of the novel metabolites identified in our analysis, including glycolytic and fatty acid oxidation intermediates, are known targets of metformin, a compound effective for diabetes therapy and prevention (Figure 4). We also identified an interesting link between DAG, a reporter metabolite for T2DM, and the CDP-choline branch of the Kennedy pathway of phospholipid metabolism (Figure 4). This pathway has been implicated in cancer development and is being established as anti-tumor drug target [56],[57]. Changes in phospholipid metabolism are known to affect the properties of cellular membranes, and subsequently signaling through membrane proteins. Further investigation of the role of phospholipids in T2DM pathogenesis may provide clues to some of the missing links that connect metabolic flux changes with insulin signaling in skeletal muscle cells.

Supplementary tables S1, S2, S3, S4 list additional reporter metabolites which are, to our knowledge, not (directly) linked with any of the known metabolic players in T2DM. Our analysis nevertheless suggests them as potential nodes of disruption or as biomarkers. Measurement of the intramyocellular concentration of the reporter metabolites in patients with diabetes risk may help to confirm the role of these metabolites in insulin resistance.

### Metabolic hubs as reporters

A particularly interesting finding from our analysis is the identification of highly connected metabolites as reporters, including ATP/ADP and NAD+/NADH. We hypothesize that diverse environmental and genetic risk factors result in insulin resistance when individuals are unable to mediate appropriate compensatory transcriptional and metabolic responses in other parts of the network connected by these hubs. Our results also suggest that alterations in gene expression linked to the highly connected co-factors are likely to be acquired features of established T2DM. Analysis of the transcriptional activity of CREB in the context of ATP concentrations and TCA cycle activity in skeletal muscle may help to elucidate regulatory mechanisms leading to these changes.

### Constraints and extension of methodology

Reconstructed human metabolic network models are still evolving, incomplete, and subject to error. Well-annotated pathways such as central carbon metabolism are thereby likely to be over-represented in the reporter analysis. In order to partially compensate for this limitation, we used two reconstructions – Recon1 and EHMN. As network reconstructions will become more complete, it will be possible to better assess the extent of this limitation. Another essential input to our algorithm, in addition to metabolic network, is gene expression data for the genes represented in the network. We would like to note that neither EHMN nor Recon1 network genes were fully represented by the microarray chips used in the two case studies (Text S1). Only 54% and 39% genes from the Recon1 and EHMN, respectively, were represented on the chips used in Mexican-American case study, while this coverage was 85% and 60% in Swedish case study. Interestingly, re-analysis of the Swedish Male dataset by using only a subset of genes from the HG-U133A chip that were represented also on the HuGeneFL (used in Mexican-American case study) showed a large overlap between the two reporter metabolite sets thus obtained (86% for T2DM *vs* NGT comparison and 69% for the rest). The details of this analysis, together with relevant statistical considerations, can be found in Text S1.

Although the present analysis identified common metabolic and regulatory signatures across the two studies, there are several differences in the study designs, and therefore the results must be regarded with certain caution. In addition to relatively low number of subjects in Mexican-American study, the differences include fasting state biopsies in Mexican-American study *vs* post insulin stimulation biopsies in Swedish study. Furthermore, the age and BMI (Body Mass Index) of the individuals participating in the two studies were different and may contribute to the differences in the observed gene expression patterns. To our knowledge, these two case studies represent the only human skeletal muscle transcriptome datasets that were available at the time of here reported computational analysis. Analysis of new datasets which may become available in the future will be useful in obtaining further insight into molecular physiology of skeletal muscle in the context of T2DM. Moreover, emergence of better or new gene expression analysis tools will help to cover parts of metabolic network that are currently inaccessible due to the lack of data.

Extension of the analysis to discover more global regulatory patterns by using additional bio-molecular interaction data [58] such as protein-DNA and protein-protein interactions will definitely be an important step in obtaining a higher resolution picture of T2DM metabolic phenotypes. Availability of such interaction data at the high confidence level of metabolic interactions is the current major bottleneck. Another essential extension of the methodology will require the use of thermodynamic data for metabolic reactions [59]–[61]. Moreover, since mRNA levels do not necessarily correlate with the protein levels, incorporation of the proteomics data together with the thermodynamic data will allow more accurate interpretation of the reporter metabolites in terms of implications for flux and concentration changes.

### Conclusions

We demonstrate the use of a network-guided data integration approach to discover key, physiologically relevant metabolic and regulatory nodes in T2DM pathogenesis. The methodology does not require the use of *a priori* disease-specific knowledge regarding the involvement of specific pathways or metabolites, thereby making it a robust and unbiased analytical framework for studying diseases linked to perturbations in the cellular metabolic network. Our results identify the highly connected metabolites ATP and NAD+ as reporters and potential mediators of the widespread changes in gene expression linked to insulin resistance in muscle. Moreover, our results extend previous knowledge about T2DM pathogenesis at the gene expression level – by reporting additional potential sites of disruption, e.g., TCA cycle and Kennedy pathway of phospholipid metabolism. Several metabolites from other pathways were also found to display significant differential gene expression of the genes around them and we suggest putative regulatory mechanisms behind these alterations. Our results suggest a framework of metabolic disruption observed with insulin resistance and diabetes, which can be used to test the role of specific pathways in mediating disease pathophysiology, and more practically, for the identification of potential biomarkers for preventive and therapeutic monitoring.

## Materials and Methods

### Gene expression and sequence data

Two datasets used in the study were obtained from the Diabetes Genome Anatomy Project website (http://www.diabetesgenome.org). Brief comparison of microarray platforms from the experimental studies [8],[9] used in the current work is presented in the Text S1. Promoter sequences for all genes were obtained from the Ensembl Biomart (http://www.ensembl.org/biomart). The transcriptional start sites (TSSs) were identified based on the annotation of the Ensembl Biomart sequences. Sequences in the −800 to 200 base pairs region of the TSS were deemed as promoter regions for the analysis. Interspersed repeats and low complexity DNA sequences were masked out.

### Metabolic networks

Two reconstructions of human metabolic network, *viz.*, Recon1 [19] and EHMN [62] were used in this study. The Homo Sapiens Recon1 is a comprehensive literature-based metabolic network reconstruction that accounts for the functions of 1496 ORFs, 2004 proteins, 2766 metabolites and 3311 metabolic and transport reactions. The ENMN (Edinburgh Human Metabolic Model) is a network reconstructed by integrating genome annotation information from different databases and metabolic reaction information from the literature. The network contains nearly 3000 metabolic reactions, which were reorganized into about 70 human-specific pathways according to their functional relationships. The two models mainly differ in the coverage of reactions and in the accounting of compartmentalization and inter-organelle transport reactions.

### Significance of differential gene expression

Preprocessing of the gene expression data was carried out by using the statistical software environment – R (www.r-project.org). The probe intensities were obtained and corrected for background by using robust multi-array average method (RMA) [63] with only perfect-match (PM) probes. Normalization was performed by using the quantiles algorithm. Gene expression values were calculated from the PM probes with the median polish summarization method [63]. All data preprocessing methods were used by invoking them through the *affy* package [64] by using *rma* function. Significance of the differential expression was calculated by using the empirical Bayes test [65]. The probe-sets were grouped into genes, and to each gene the differential expression was defined by choosing the value from the top level probe-set (using the probe-set rank defined by Affymetrix). In case of more than one probe-set present at the top level, the median value was used.

### Reporter metabolites

Each metabolite in the metabolic network was scored based on the scores of its *k* neighbor enzymes (i.e. enzymes catalyzing reactions involving that metabolite, either as a substrate or as a product). Each enzyme was assigned with a p-value for differential expression based on the p-value of the gene encoding for that enzyme. In case of isozymes and enzyme-complexes, genes with most significant expression change were used to score the enzyme (Figure 1). P-values of genes *p_i_*, indicating the significance of differential expression, were converted to Z-scores *Z_i_* by using the inverse normal cumulative distribution function (CDF) (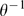): 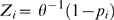. All metabolite nodes were assigned a Z-score, *Z_metabolit_*_e_, calculated as aggregated Z scores of the *k* neighbor enzymes: 
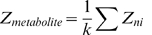
. *Z_metabolite_* scores were then corrected for the background distribution by subtracting the mean (*μ_k_*) and dividing by the standard deviation (*σ_k_*) of the aggregated Z scores derived by sampling 10000 sets of *k* enzymes from the network: 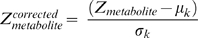. Corrected Z-scores were then transformed to p-values by using CDF. Metabolites with p-values less than 0.05 were deemed as reporter metabolites. Detailed information on the reporter scoring can be found in the Text S1 and [14].

### Transcription factor binding site enrichment

For all reporter metabolites, we assessed enrichment of known protein-binding sequence motifs in the promoter regions (−800 to 200 base pairs relative to the transcription start site) of the corresponding neighbor genes. In order to obtain robust results, we only considered sets consisting of at least 5 up- or down-regulated genes. For each reporter metabolite, the sequences of all enzyme neighbors were used as the positive sequence set, whereas all other enzymes in the network model were used as the negative (background) set. Known motifs were identified by using position frequency matrices of all known motifs stored in the TRANSFAC database [66]. The motif enrichment analysis tool ASAP [67] was used to scan all TRANSFAC motif matrices against the positive sequence sets of each reporter metabolite. The negative sequence sets were used together with 2^nd^ order background model. A one-tailed Fisher's exact test was used to assess per-sequence over-representation of any known motif, and the threshold used to calculate significance for each TRANSFAC matrix was set to 70% of the highest-scoring sequence motif. The q-value cut-off criteria [29] was used as a post-data measure of statistical significance of all motifs found to be significantly enriched.

## Supporting Information

Text S1 Supporting text describing scoring methodology and datasets.
(0.74 MB PDF)

Table S1 Reporter metabolites for Swedish male dataset (Recon1).
(0.05 MB XLS)

Table S2 Reporter metabolites for Swedish male dataset (EHMN).
(0.07 MB XLS)

Table S3 Reporter metabolites for Mexican-American dataset (Recon1).
(0.03 MB XLS)

Table S4 Reporter metabolites for Mexican-American dataset (EHMN).
(0.05 MB XLS)

Table S5 Overlapping reporter metabolites between two case studies (Recon1).
(0.05 MB XLS)

Table S6 Overlapping reporter metabolites between two case studies (EHMN).
(0.06 MB XLS)

Table S7 Results of the motif enrichment analysis.
(0.03 MB PDF)

Table S8 Experimentally studies linking metabolite levels to T2DM pathophysiology
(0.12 MB PDF)

Figure S1 Hierarchical clustering of pair-wise comparisons within the Swedish male and Mexican-American datasets based on the overlapping reporter metabolites (EHMN network).
(0.21 MB TIF)

## References

[1] Zimmet P, Alberti KG, Shaw J (2001). Global and societal implications of the diabetes epidemic.. Nature.

[2] Simpson RW, Shaw JE, Zimmet PZ (2003). The prevention of type 2 diabetes–lifestyle change or pharmacotherapy? A challenge for the 21st century.. Diabetes Res Clin Pract.

[3] Shulman GI (2000). Cellular mechanisms of insulin resistance.. J Clin Invest.

[4] Pehling G, Tessari P, Gerich JE, Haymond MW, Service FJ (1984). Abnormal meal carbohydrate disposition in insulin-dependent diabetes. Relative contributions of endogenous glucose production and initial splanchnic uptake and effect of intensive insulin therapy.. J Clin Invest.

[5] Muoio DM, Newgard CB (2008). Molecular and metabolic mechanisms of insulin resistance and [beta]-cell failure in type 2 diabetes.. Nat Rev Mol Cell Biol.

[6] Saltiel AR, Kahn CR (2001). Insulin signalling and the regulation of glucose and lipid metabolism.. Nature.

[7] Kelley DE, He J, Menshikova EV, Ritov VB (2002). Dysfunction of mitochondria in human skeletal muscle in type 2 diabetes.. Diabetes.

[8] Mootha VK, Lindgren CM, Eriksson KF, Subramanian A, Sihag S (2003). PGC-1alpha-responsive genes involved in oxidative phosphorylation are coordinately downregulated in human diabetes.. Nat Genet.

[9] Patti ME, Butte AJ, Crunkhorn S, Cusi K, Berria R (2003). Coordinated reduction of genes of oxidative metabolism in humans with insulin resistance and diabetes: Potential role of PGC1 and NRF1.. Proc Natl Acad Sci U S A.

[10] Boden G (1996). Fatty acids and insulin resistance.. Diabetes Care.

[11] Ueki K, Kondo T, Tseng YH, Kahn CR (2004). Central role of suppressors of cytokine signaling proteins in hepatic steatosis, insulin resistance, and the metabolic syndrome in the mouse.. Proc Natl Acad Sci U S A.

[12] Sreekumar R, Halvatsiotis P, Schimke JC, Nair KS (2002). Gene Expression Profile in Skeletal Muscle of Type 2 Diabetes and the Effect of Insulin Treatment..

[13] Yang X, Pratley RE, Tokraks S, Bogardus C, Permana PA (2002). Microarray profiling of skeletal muscle tissues from equally obese, non-diabetic insulin-sensitive and insulin-resistant Pima Indians.. Diabetologia.

[14] Patil KR, Nielsen J (2005). Uncovering transcriptional regulation of metabolism by using metabolic network topology.. Proc Natl Acad Sci U S A.

[15] Seggewiss J, Becker K, Kotte O, Eisenacher M, Yazdi MR (2006). Reporter metabolite analysis of transcriptional profiles of a Staphylococcus aureus strain with normal phenotype and its isogenic hemB mutant displaying the small-colony-variant phenotype.. J Bacteriol.

[16] David H, Hofmann G, Oliveira AP, Jarmer H, Nielsen J (2006). Metabolic network driven analysis of genome-wide transcription data from Aspergillus nidulans.. Genome Biol.

[17] Capel F, Klimcakova E, Viguerie N, Roussel B, Vitkova M (2009). Macrophages and adipocytes in human obesity: adipose tissue gene expression and insulin sensitivity during calorie restriction and weight stabilization.. Diabetes.

[18] Baxter CJ, Redestig H, Schauer N, Repsilber D, Patil KR (2007). The metabolic response of heterotrophic Arabidopsis cells to oxidative stress.. Plant Physiol.

[19] Duarte NC, Becker SA, Jamshidi N, Thiele I, Mo ML (2007). Global reconstruction of the human metabolic network based on genomic and bibliomic data.. Proc Natl Acad Sci U S A.

[20] Ma H, Sorokin A, Mazein A, Selkov A, Selkov E (2007). The Edinburgh human metabolic network reconstruction and its functional analysis.. Mol Syst Biol.

[21] Roden M (2005). Muscle triglycerides and mitochondrial function: possible mechanisms for the development of type 2 diabetes.. Int J Obes (Lond).

[22] Savage DB, Petersen KF, Shulman GI (2007). Disordered lipid metabolism and the pathogenesis of insulin resistance.. Physiol Rev.

[23] Holland WL, Brozinick JT, Wang LP, Hawkins ED, Sargent KM (2007). Inhibition of ceramide synthesis ameliorates glucocorticoid-, saturated-fat-, and obesity-induced insulin resistance.. Cell Metab.

[24] Chavez JA, Summers SA (2003). Characterizing the effects of saturated fatty acids on insulin signaling and ceramide and diacylglycerol accumulation in 3T3-L1 adipocytes and C2C12 myotubes.. Arch Biochem Biophys.

[25] Salek RM, Maguire ML, Bentley E, Rubtsov DV, Hough T (2007). A metabolomic comparison of urinary changes in type 2 diabetes in mouse, rat, and human.. Physiol Genomics.

[26] Newgard CB, An J, Bain JR, Muehlbauer MJ, Stevens RD (2009). A Branched-Chain Amino Acid-Related Metabolic Signature that Differentiates Obese and Lean Humans and Contributes to Insulin Resistance.. Cell Metabolism.

[27] Fleischman A, Kron M, Systrom DM, Hrovat M, Grinspoon SK (2009). Mitochondrial Function and Insulin Resistance in Overweight and Normal-Weight Children.. J Clin Endocrinol Metab.

[28] Phielix E, Schrauwen-Hinderling VB, Mensink M, Lenaers E, Meex R (2008). Lower intrinsic ADP-stimulated mitochondrial respiration underlies in vivo mitochondrial dysfunction in muscle of male type 2 diabetic patients.. Diabetes.

[29] Storey JD, Tibshirani R (2003). Statistical significance for genomewide studies.. Proc Natl Acad Sci U S A.

[30] Bajaj M, Defronzo RA (2003). Metabolic and molecular basis of insulin resistance.. J Nucl Cardiol.

[31] Itani SI, Ruderman NB, Schmieder F, Boden G (2002). Lipid-induced insulin resistance in human muscle is associated with changes in diacylglycerol, protein kinase C, and IkappaB-alpha.. Diabetes.

[32] Patti ME, Corvera S (2009). The Role of Mitochondria in the Pathogenesis of Type 2 Diabetes.. Endo Reviews: In press.

[33] Koves TR, Li P, An J, Akimoto T, Slentz D (2005). Peroxisome proliferator-activated receptor-gamma co-activator 1alpha-mediated metabolic remodeling of skeletal myocytes mimics exercise training and reverses lipid-induced mitochondrial inefficiency.. J Biol Chem.

[34] Kersten S, Desvergne B, Wahli W (2000). Roles of PPARs in health and disease.. Nature.

[35] Sen CK, Packer L (1996). Antioxidant and redox regulation of gene transcription.. Faseb J.

[36] Scarpulla RC (2008). Nuclear control of respiratory chain expression by nuclear respiratory factors and PGC-1-related coactivator.. Ann N Y Acad Sci.

[37] Scarpulla RC (2006). Nuclear control of respiratory gene expression in mammalian cells.. J Cell Biochem.

[38] Szendroedi J, Schmid AI, Chmelik M, Toth C, Brehm A (2007). Muscle mitochondrial ATP synthesis and glucose transport/phosphorylation in type 2 diabetes.. PLoS Med.

[39] Petersen KF, Dufour S, Shulman GI (2005). Decreased insulin-stimulated ATP synthesis and phosphate transport in muscle of insulin-resistant offspring of type 2 diabetic parents.. PLoS Med.

[40] Petersen KF, Befroy D, Dufour S, Dziura J, Ariyan C (2003). Mitochondrial dysfunction in the elderly: possible role in insulin resistance.. Science.

[41] Ristow M, Zarse K, Oberbach A, Kloting N, Birringer M (2009). Antioxidants prevent health-promoting effects of physical exercise in humans.. Proc Natl Acad Sci U S A.

[42] Brekasis D, Paget MS (2003). A novel sensor of NADH/NAD+ redox poise in Streptomyces coelicolor A3(2).. Embo J.

[43] Zhang Q, Piston DW, Goodman RH (2002). Regulation of corepressor function by nuclear NADH.. Science.

[44] Lin SJ, Defossez PA, Guarente L (2000). Requirement of NAD and SIR2 for life-span extension by calorie restriction in Saccharomyces cerevisiae.. Science.

[45] Anderson RM, Latorre-Esteves M, Neves AR, Lavu S, Medvedik O (2003). Yeast life-span extension by calorie restriction is independent of NAD fluctuation.. Science.

[46] Rutter J, Reick M, Wu LC, McKnight SL (2001). Regulation of clock and NPAS2 DNA binding by the redox state of NAD cofactors.. Science.

[47] Agarwal AK, Auchus RJ (2005). Minireview: cellular redox state regulates hydroxysteroid dehydrogenase activity and intracellular hormone potency.. Endocrinology.

[48] Rodgers JT, Lerin C, Haas W, Gygi SP, Spiegelman BM (2005). Nutrient control of glucose homeostasis through a complex of PGC-1alpha and SIRT1.. Nature.

[49] Cimini D, Patil KR, Schiraldi C, Nielsen J (2009). Global transcriptional response of Saccharomyces cerevisiae to the deletion of SDH3.. BMC Syst Biol.

[50] Raghevendran V, Patil KR, Olsson L, Nielsen J (2006). Hap4 is not essential for activation of respiration at low specific growth rates in Saccharomyces cerevisiae.. J Biol Chem.

[51] Schuurmans JM, Rossell SL, van Tuijl A, Bakker BM, Hellingwerf KJ (2008). Effect of hxk2 deletion and HAP4 overexpression on fermentative capacity in Saccharomyces cerevisiae.. FEMS Yeast Res.

[52] He J, Watkins S, Kelley DE (2001). Skeletal muscle lipid content and oxidative enzyme activity in relation to muscle fiber type in type 2 diabetes and obesity.. Diabetes.

[53] Daran-Lapujade P, Rossell S, van Gulik WM, Luttik MA, de Groot MJ (2007). The fluxes through glycolytic enzymes in Saccharomyces cerevisiae are predominantly regulated at posttranscriptional levels.. Proc Natl Acad Sci U S A.

[54] Bruning T, Vamvakas S, Makropoulos V, Birner G (1998). Acute intoxication with trichloroethene: clinical symptoms, toxicokinetics, metabolism, and development of biochemical parameters for renal damage.. Toxicol Sci.

[55] Laughter AR, Dunn CS, Swanson CL, Howroyd P, Cattley RC (2004). Role of the peroxisome proliferator-activated receptor alpha (PPARalpha) in responses to trichloroethylene and metabolites, trichloroacetate and dichloroacetate in mouse liver.. Toxicology.

[56] Ramirez de Molina A, Gallego-Ortega D, Sarmentero J, Banez-Coronel M, Martin-Cantalejo Y (2005). Choline kinase is a novel oncogene that potentiates RhoA-induced carcinogenesis.. Cancer Res.

[57] Banez-Coronel M, de Molina AR, Rodriguez-Gonzalez A, Sarmentero J, Ramos MA (2008). Choline kinase alpha depletion selectively kills tumoral cells.. Curr Cancer Drug Targets.

[58] Oliveira AP, Patil KR, Nielsen J (2008). Architecture of transcriptional regulatory circuits is knitted over the topology of bio-molecular interaction networks.. BMC Syst Biol.

[59] Cakir T, Patil KR, Onsan Z, Ulgen KO, Kirdar B (2006). Integration of metabolome data with metabolic networks reveals reporter reactions.. Mol Syst Biol.

[60] Kummel A, Panke S, Heinemann M (2006). Putative regulatory sites unraveled by network-embedded thermodynamic analysis of metabolome data.. Mol Syst Biol.

[61] Henry CS, Broadbelt LJ, Hatzimanikatis V (2007). Thermodynamics-based metabolic flux analysis.. Biophys J.

[62] Ma H, Goryanin I (2008). Human metabolic network reconstruction and its impact on drug discovery and development.. Drug Discov Today.

[63] Irizarry RA, Hobbs B, Collin F, Beazer-Barclay YD, Antonellis KJ (2003). Exploration, normalization, and summaries of high density oligonucleotide array probe level data.. Biostatistics.

[64] Gautier L, Cope L, Bolstad BM, Irizarry RA (2004). affy–analysis of Affymetrix GeneChip data at the probe level.. Bioinformatics.

[65] Smyth GK (2004). Linear models and empirical bayes methods for assessing differential expression in microarray experiments.. Stat Appl Genet Mol Biol.

[66] Matys V, Fricke E, Geffers R, Gossling E, Haubrock M (2003). TRANSFAC: transcriptional regulation, from patterns to profiles.. Nucleic Acids Res.

[67] Marstrand TT, Frellsen J, Moltke I, Thiim M, Valen E (2008). Asap: a framework for over-representation statistics for transcription factor binding sites.. PLoS ONE.

