# Supplementary material for: Metabolic Network Topology Reveals Transcriptional Regulatory Signatures of Type 2 Diabetes
Source: PLoS Comput Biol. 2010 Apr 1;6(4):e1000729. doi: 10.1371/journal.pcbi.1000729 (PMC2848542; doi:10.1371/journal.pcbi.1000729)
Supplement: Text S1 — Supporting text describing scoring methodology and datasets. (0.74 MB PDF) [file pcbi.1000729.s001.pdf]

Supporting Text S1

From gene expression to reporter metabolites – calculation of reporter metabolite score

We present a step-by-step procedure for calculation of reporter metabolites. The first step is to estimate p-value for the significance of differential expression for all of the genes across two phenotypes in question, for example, T2DM *vs.* NGT. In our analysis we used empirical Bayes test [1] to assess the significance of differentially expressed genes. It is possible to use other statistical methods, e.g. t-test or ranksum test. The choice of the method depends on the underlying structure of the data and assumptions that can be made. The statistical test assigns a *p*-value to each of the probe sets by taking into account the variation within the groups being compared. A gene is usually represented by several probe sets, and in such case a *p*-value for differentially expressed gene is defined by choosing the value from the top probe-set[1], according to the probe-set ranks (probe-set ranks are defined by the manufacturer, see Affymetrix web site-http://www.affymetrix.com/). Further, by using inverse normal cumulative distribution function, the *p*-value[2] of each of the probe set (gene) is converted to a standard Z-score with a mean of 0 and a variance of 1, thus uniformly distributed *p*-values are transformed to a normally distributed random variable (Figure SM1).

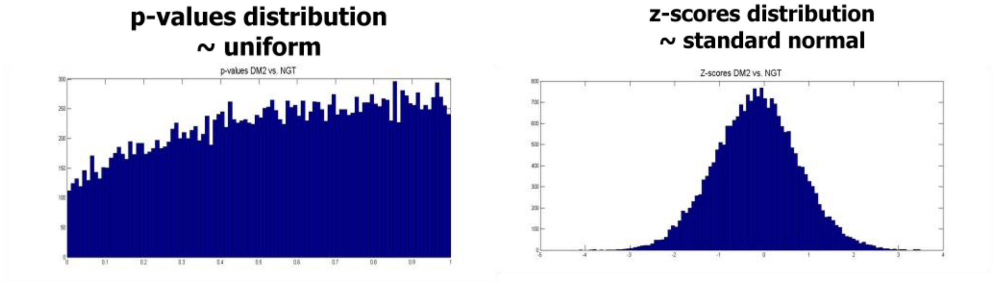

Figure SM1. *Conversion of gene p-values to standard Z-score using inverse cumulative function*

The next step is to use gene Z-scores to assign Z-score to the metabolites. Z-score of a metabolite is an average of Z-scores of genes connected to that metabolite in the metabolic model. In order to evaluate the significance of metabolite scores, average Z-score of each of the metabolites needs to be corrected for the background Z-score distribution[3] (see Figure 6 in [2]).

After background correction each metabolite's Z-score is converted to a *p*-value by using normal cumulative distribution function. Overall procedure including a calculation example is illustrated in Figure SM2. Metabolites having significant *p*-values are termed reporter metabolites – metabolites around which the most significant transcriptional changes occur. For readers interested in further details on the scoring system, including alternative scoring schemes and background distribution, please refer to Oliveira *et al.* (BMC Systems Biology, 2008).

---

[1] In case if there are several probe-sets representing the same rank the median *p*-value is selected
[2] $Z = cdf^{-1}(1 - p\_value)$ - in MS Excel use **NORMSINV(p)**, in MATLAB **norminv(p,mu,sigma)**
[3] See main text, materials and methods section for background distribution estimation.

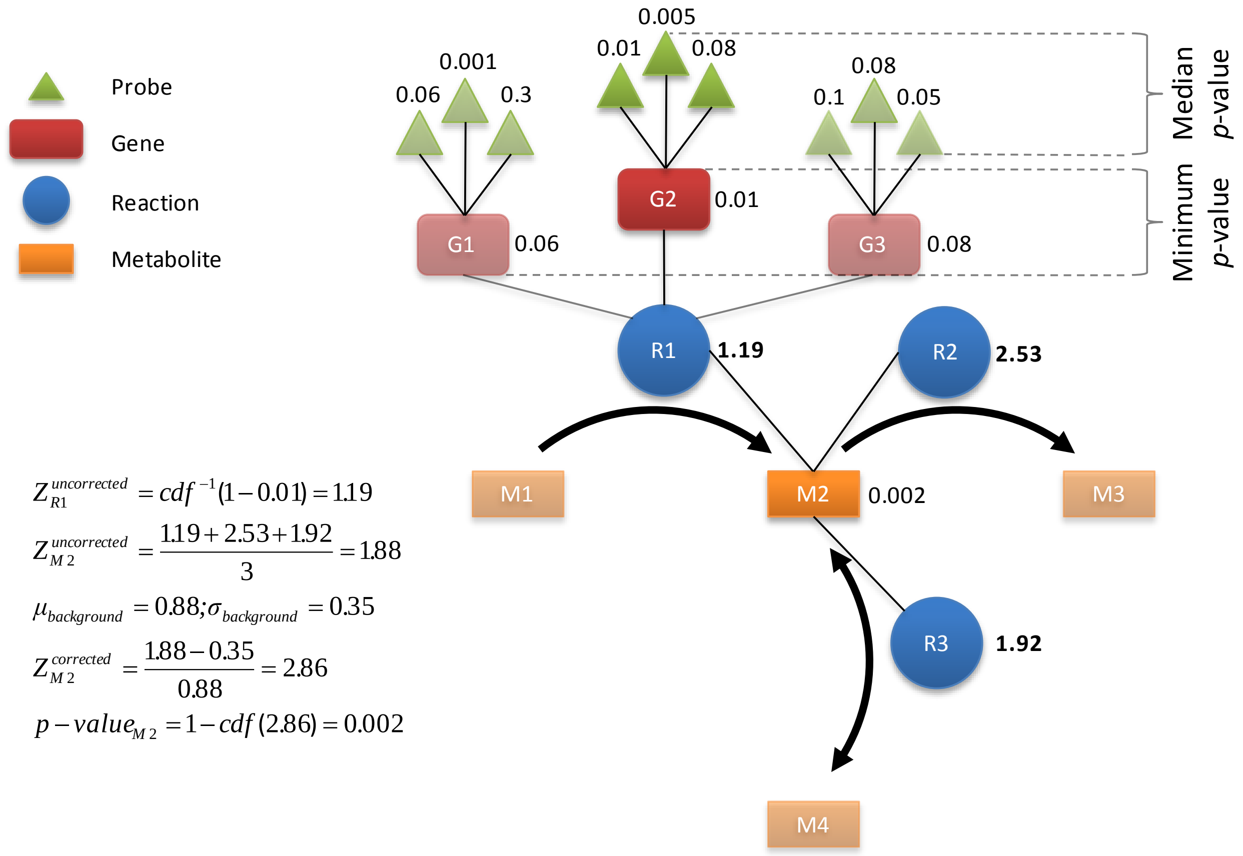

Figure SM2. Scoring system for identification of reporter metabolites. Each metabolite is scored based on the scores of the associated enzyme-catalyzed reactions. Each enzyme, in turn, is assigned a score based on median of the p-values of the probes representing the corresponding gene. In case of a reaction catalyzed by an enzyme complex or a set of isozymes, minimum of the p-values of the corresponding enzymes is chosen. Numbers in bold are Z-scores for each reaction, the rest of the numbers represent p-values (significance of differential expression).

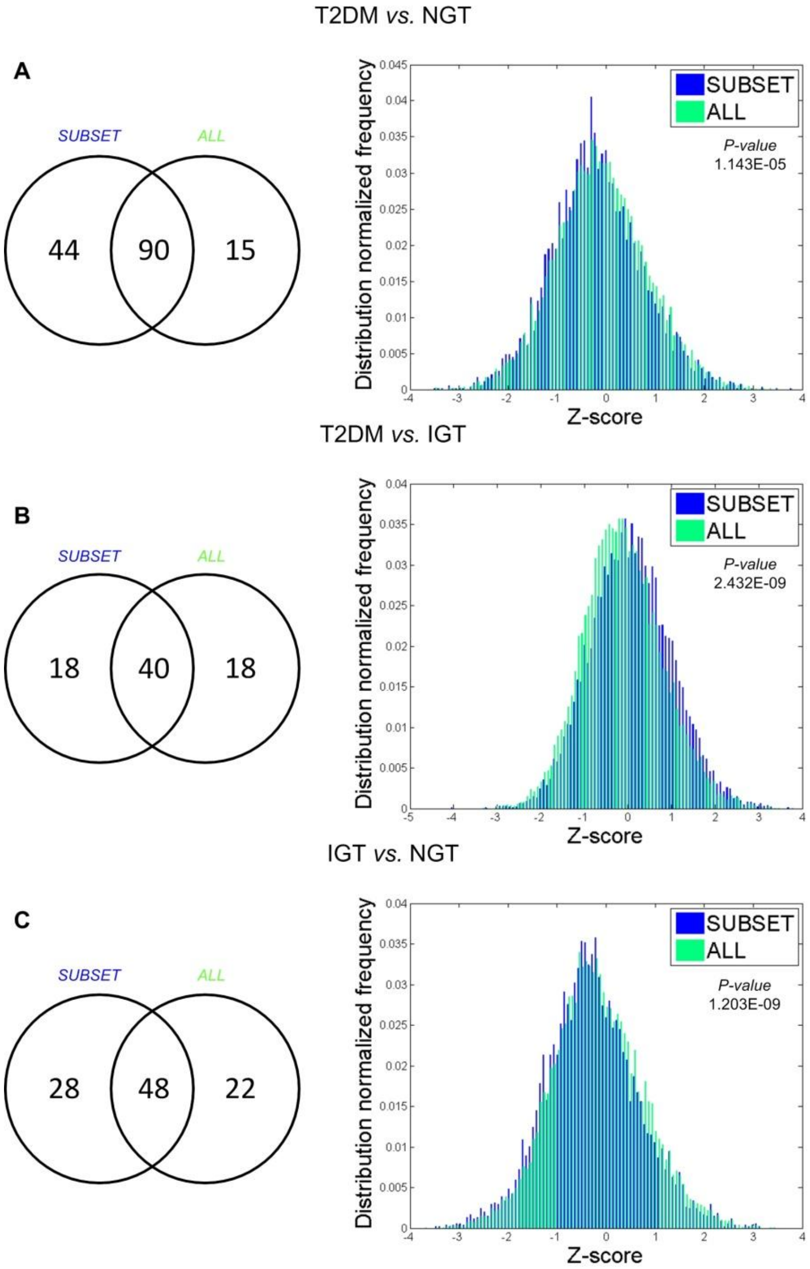

Figure SM3. Comparison of results from reporter metabolite analysis based on two different sets of metabolic genes – one being proper subset of the other. In order to assess the effect of relatively low coverage of metabolic genes on the HuGeneFL (used in Mexican-American case study), we re-analyzed the Swedish Male dataset by using only a subset of genes from the HG-U133A chip that were represented also on the HuGeneFL. The results show large overlap between the two reporter metabolite sets thus obtained. The left panel shows Venn diagram illustrating the overlap between the two reporter metabolite sets, while the right hand side panel shows the distribution of Z-scores for the two metabolic gene sets. P-values shown are results of Student's t-test comparing the two distributions. In all comparisons, p-values are very low, implying that the two distributions are distinct. This is one of the contributing factors to the difference between the reporter metabolite results, in addition to the fact that the number of neighbors for several metabolites is also different for each gene subsets. For mathematical description of the relationship between these two factors and reporter score, please see materials and methods section in the main text.

Table SM1. *Brief comparison of the two experimental studies used for identifying metabolic and regulatory signatures of T2DM.*

	**Swedish male dataset** [3]			**Mexican-American dataset** [4]		
	**NGT**	**IGT**	**DM2**	**FH-**	**FH+**	**DM2**
**Ethnicity**	Caucasian			Mexican American		
**Subject Number**	17	8	18	6	4	5
**Age**	66.1 (3.4)	66.4 (1.6)	65.5 (1.8)	38.5 (3.7)	40.8 (2.6)	43.8 (2.1)
**BMI, kg/m^2**	23.6 (3.4)	27.1 (4.8)	27.3 (4.0)	31.2 (0.8)	28.9 (1.4)	37.4 (5.8)
**Microarray**	Affymetrix HG-U133A			Affymetrix HuGeneFL		

Table SM2. *Brief comparison of microarray platforms from experimental studies used for identifying metabolic and regulatory signatures of T2DM.*

**Microarray platform**	HG – U133A [5]	HuGeneFL (Hu6800) [5]
**Number of genes**	>14 500	~6800
**Number of probe sets**	>22 000	6940
**Recon1 coverage*, %**	85%	54%
**EHMN coverage*, %**	60%	39%

*Percentage of genes from the model represented on the microarray chip

References

1. Smyth GK (2004) Linear models and empirical bayes methods for assessing differential expression in microarray experiments. Stat Appl Genet Mol Biol 3: Article3.
2. Oliveira AP, Patil KR, Nielsen J (2008) Architecture of transcriptional regulatory circuits is knitted over the topology of bio-molecular interaction networks. BMC Syst Biol 2: 17.
3. Mootha VK, Lindgren CM, Eriksson KF, Subramanian A, Sihag S, et al. (2003) PGC-1alpha-responsive genes involved in oxidative phosphorylation are coordinately downregulated in human diabetes. Nat Genet 34: 267-273.
4. Patti ME, Butte AJ, Crunkhorn S, Cusi K, Berria R, et al. (2003) Coordinated reduction of genes of oxidative metabolism in humans with insulin resistance and diabetes: Potential role of PGC1 and NRF1. Proc Natl Acad Sci U S A 100: 8466-8471.
