# Supplementary material for: Metabolic Network Topology Reveals Transcriptional Regulatory Signatures of Type 2 Diabetes
Source: PLoS Comput Biol. 2010 Apr 1;6(4):e1000729. doi: 10.1371/journal.pcbi.1000729 (PMC2848542; doi:10.1371/journal.pcbi.1000729)
Supplement: Table S7 — Results of the motif enrichment analysis. (0.03 MB PDF) [file pcbi.1000729.s008.pdf]

Table S7: Motif results for one comparison for case-1 and one for case-2

Reporter Metabolites	T2 DM / FH- Down	T2DM / FH- Up	T2DM NGT Down	T2DM / NGT Up
**ADP[c]**	DEAF1; GAL4; HEN1; LUN-1; p53; Pax-1; Roaz; Tax/CREB	ABF1; APOLYA; Arnt; Bel-1; DEAF1; Dfd; HIF-1; HTF; MYB.Ph3; Pax-4; Pax-6; Pax-9; S8; ste11	GCN4; HEN1; HSF; Nrf-1; p53; STAT; STAT1; STAT3	E2F; GAL4; Nkx6-2; O2; Pax-9; PPARalpha:RXR-alpha; Tax/CREB; UF1H3BETA
**ATP[c]**	LUN-1; p53; PPARG; Roaz; Tax/CREB	-	-	-
**2-Oxoglutarate[m]**	-	Athb-1; Cart-1; FOXJ2; LAC9; Lhx3; LUN-1; MEF-2; myogenin / NF-1; POU3F2; R; v-Jun	-	-
**L-Alanine[c]**	-	-	-	ABF1; ABF1; PPARalpha:RXR-alpha; PPARG
**AMP[c]**	-	-	AR; CRP; Lhx3; NKX6-1; POU3F2; RSRFC4; SRF; STAT1; STAT5A (homotetramer)	ATATA; bZIP910; bZIP911
**ATP[c]**	-	ABF1; APOLYA; Arnt; Brn-2; c-Myc:Max; DEAF1; Dfd; E74A; HIF-1; Max; MYB.Ph3; ste11; v-Myb; XFD-2	-	bZIP911; LEU3; MEF-2; NF-kappaB; Nkx6-2; Pax-9 ; PPARalpha:RXR-alpha; Tax/CREB; UF1H3BETA
**CO2[m]**	-	DEAF1; LUN-1; TCF11:MafG; XBP-1	-	-
**Coenzyme A[m]**	-	Alx-4; CDP; Croc; DEAF1; DREF; Evi-1; HSF; POU3F2	-	-
**D-Fructose 6-phosphate[c]**	-	ABF1; LUN-1; LXR direct repeat 4; NAC69-1; STAT1; STAT3	GAL4; NAC69-1; STAT; STAT3	Clox; GR; ISRE; PPARG; v-Maf
**L-Glutamate[m]**	-	LUN-1; NRSE; Tax/CREB	-	-
**H+[c]**	ABDA; FAC1; p53; STAT	ABF1; AGL3; AIRE; aMEF-2; ANTP; AR; Arnt;	-	-

		ATHB-5; ATHB-9; Bcd; BR-C Z1; BR-C Z2; BR-C Z4; c-Maf; C/EBPgamma; Cdx-2; CEBP; ces-2; D-Type LTRs; Dfd; dri; Egr-2; Elf-1; EmBP-1b Evi-1; FAC1; OXD3; FOXJ2; Freac-7; Ftz; FXR/RXR-alpha; Gfi-1; HAHB-4; HAP2/3/4; Hb; HFH-1; HFH-3; HFH-4; HFH-8; HLF; HMGIY; HNF-1; HNF-3beta; HNF-4; HNF-6; HSF; HTF; ICSBP; ID1; IPF1; KNI; Lentiviral Poly A; Lhx3; MADS-B; MEF-2; MEIS1A:HOXA; MEIS1B:HOXA; MIG1; MYB.Ph3; Nkx2-5; NKX3A; Nkx6-2; O2; Ovo; Pax-4; PEND; PITX2; POU3F2; RSRFC4; S8; SBF-1; Skn-1; SOX; Sox-5; SRF; SRY; STAT3; ste11; TAF; TATA; TBP; TCF;cTGA1a; v-Myb; WRKY; XFD-1;cXFD-2; Zec		
**H+[m]**	ABF1; AIRE; CDP; Clox; CRP; Evi-1; FOXP1; HNF-1;HNF1;MCM1+SFF; MEF-2; NKX6-1; PLZF; POU3F2;RSRFC4	ABF1; aMEF-2; BR-C Z2; Cart-1; ces-2; CF1A; CF2-II; Croc; Dfd; DREF; Evi-1; FOXJ2; Freac-7; HAHB-4; HFH-1; HFH-3; HFH-4; HFH-8; HNF-1; HNF-6; HSF; HTF; Lhx3; Max; MCM1+SFF;	-	-

		NKX3A; OCT-x; Pax-1; POU3F2; POU6F1; R; RSRFC4; S8; STE11; Tax/CREB; TCF; v-Myb; XFD-2; XFD-3		
**Bicarbonate[c]**	-	-	-	GAL4; NRSF
**Bicarbonate[e]**	-	-	-	GAL4; NRSF
**Methylglyoxal[c]**	-	-	-	DEAF1; IRF-2; ISRE; SRF
**Nicotinamide adenine dinucleotide[c]**	-	AR; Brachyury; HOXA5 (Hox-1.3); HSF; O2; PPARG; SRF	-	-
**Nicotinamide Adenine dinucleotide[m]**	ABF1; Alx-4; NAC69-1; SEF-1; STAT	AGL15; AGL2; AGL3; AhR; AIRE; aMEF-2; ATATA; BR-C Z1; BR-C Z2; BR-C Z3; Cart-1; CDP; CF2-II; Croc; DEAF1; E4BP4; FOXJ2; FOXO1; Freac-2; Freac-3; Freac-7; Gfi-1; GR; HFH-4; HNF-1; HNF-3beta; HNF-6; HSF1; HTF; IRF-1; Lentiviral TATA; Lhx3; MADS-A; MEF-2; NKX3A; OCT-x; p53; POU3F2; POU6F1; R; RSRFC4; S8; SOX; ste11; TEF; XFD-2; XFD-3	-	-
**Ammonium[c]**	-	-	-	ABF1; AFP1; AIRE; AR; Cart-1; DAF-16; Evi-1; GAL4; GCN4; GR; IRF-2; LAC9; Lhx3; LUN-1; MYB.Ph3; PLZF; POU6F1; R; SREBP-1
**Phosphatidylinositol (homo**	-	ABF1; APOLYA; AR; ATHB-9; Bel-1;	-	Evi-1; MADS-A; Mat1-Mc; NRSF;

**sapiens)[c]**		Cart-1; CDP; CDP CR3; Clox; CRP; DEAF1; Evi-1; FOXP1; MADS-A; MCM1+SFF; MIF-1; MYB.Ph3; O2; p53; Pax-1; Pax-4; Pax-6; Pbx1b; PLZF; POU3F2; PPARG; SRF; STAT; STAT1; TCF11:MafG; Zec		p53; PPARG; STAT
**Phosphatidylinositol 4,5-bisphosphate (Homo sapiens)[c]**	-	-	-	AR; Brachyury; CDP CR3; GCNF; O2; p53; PPARG; SEF-1; SRF; v-Jun
**Phosphate[c]**	-	-	ABF1; AR; HEN1; NAC69-1; p53; Roaz; SEF-1	ATHB-5; Brn-2; ces-2; CF2-II; CRE-BP1; GAL4; Hb; HNF-4alpha1; HSF; MYB80; O2; p53; STAT5A (homodimer); STAT5B (homodimer); TAF; Tax/CREB; UF1H3BETA; ZNF219
**Phosphate[c]**	-	D-Type LTRs; Evi-1; FOXJ2; SEF-1; v-Maf	-	-
**Pyruvate[m]**	-	ABF1; GAL4	-	-
**Pyruvate[m]**	-	ABF1; CDP CR3; GAL4; HSF; PPAR	-	-
**Succinyl-CoA[c]**	-	Alx-4; Athb-1; Bach1; Cart-1; CRP; DEAF1; Evi-1; LUN-1; O2; Pax-1; Pax-4; POU3F2; PPAR; PPARalpha:RXR-alpha; STAT; STAT5A (homotetramer)	-	-
