# Supplementary material for: Metabolic Network Topology Reveals Transcriptional Regulatory Signatures of Type 2 Diabetes
Source: PLoS Comput Biol. 2010 Apr 1;6(4):e1000729. doi: 10.1371/journal.pcbi.1000729 (PMC2848542; doi:10.1371/journal.pcbi.1000729)
Supplement: Table S8 — Experimentally studies linking metabolite levels to T2DM pathophysiology (0.12 MB PDF) [file pcbi.1000729.s009.pdf]

Table S8. Experimentally studies linking metabolite levels to T2DM pathophysiology.

Reference	Metabolites	Organism	Source	Phenotypic observation
Koves et al. [1]	Citrate; L-Malate; Fumarate; L-Lactate; 2-Oxoglutarate	Rodent	Muscle	Lower amounts of organic acids were found in obese diabetic rats compared to the lean diabetic rats
Salek et al. [2]	L-Leucine; Pyruvate; Citrate; beta-Alanine; D-Alanine; L-Alanine	Human	Urine	Increased amounts in T2DM
	L-Malate;, L-Glutamate; Fumarate; Phosphocreatine			Decreased amounts in T2DM
Ellis et al [3]<br>Ye et al [4]	Palmitoyl-CoA; Tetradecanoyl-CoA; Palmitoleoyl-CoA; Decanoyl-CoA; Lauroyl-CoA	Human/Rodent	Muscle	Strong negative correlation between insulin sensitivity and the content of LC-CoA (Ellis et al)<br>Fall of glucose uptake into muscle was accompanied by increase in plasma TG and muscle LC-CoA (Ye et al)
Chavez et al [5]	1,2 -Diacyl-sn-glycerol (DAG); Palmitoyl-CoA; Palmitoleoyl-CoA	Rodent tissue culture	Adipocytes/myotubes	Palmitate induced the accrual of ceramide and diacylglycerol (DAG), two lipid metabolites inhibit insulin signaling in cultured cells
Neschen et al [6]	Glycerol -3-phosphate; glycerol	Rodent	Liver	Suppression of mitochondrial GPAT1 (TG synthesis) activity results in lowered DAG and reversal of hepatic insulin resistance
Yu et al. [7]	1,2 -Diacyl-sn-glycerol (DAG); Palmitoyl-CoA; Palmitoleoyl-CoA	Rodent	Muscle	Accumulation of DAG and other bioactive lipid molecules is thought to engage stress-activated serine kinases that interfere with insulin signal transduction
Holland et all [8]	Palmitoleoyl-CoA	Rodent	Liver	Inhibition of ceramide synthesis protected rodents against insulin resistance induced by infusion of saturated fatty acid

References

1. *Koves TR, Ussher JR, Noland RC, Slentz D, Mosedale M, et al. (2008) Mitochondrial overload and incomplete fatty acid oxidation contribute to skeletal muscle insulin resistance. Cell Metab 7: 45-56.*

2. *Salek RM, Maguire ML, Bentley E, Rubtsov DV, Hough T, et al. (2007) A metabolomic comparison of urinary changes in type 2 diabetes in mouse, rat, and human. Physiol Genomics 29: 99-108.*

3. *Ellis BA, Poynten A, Lowy AJ, Furler SM, Chisholm DJ, et al. (2000) Long-chain acyl-CoA esters as indicators of lipid metabolism and insulin sensitivity in rat and human muscle. Am J Physiol Endocrinol Metab 279: E554-560.*

4. *Ye JM, Doyle PJ, Iglesias MA, Watson DG, Cooney GJ, et al. (2001) Peroxisome proliferator-activated receptor (PPAR)-alpha activation lowers muscle lipids and improves insulin sensitivity in high fat-fed rats: comparison with PPAR-gamma activation. Diabetes 50: 411-417.*

5. *Chavez JA, Summers SA (2003) Characterizing the effects of saturated fatty acids on insulin signaling and ceramide and diacylglycerol accumulation in 3T3-L1 adipocytes and C2C12 myotubes. Arch Biochem Biophys 419: 101-109.*

6. *Neschen S, Morino K, Hammond LE, Zhang D, Liu ZX, et al. (2005) Prevention of hepatic steatosis and hepatic insulin resistance in mitochondrial acyl-CoA:glycerol-sn-3-phosphate acyltransferase 1 knockout mice. Cell Metab 2: 55-65.*

7. *Yu C, Chen Y, Cline GW, Zhang D, Zong H, et al. (2002) Mechanism by which fatty acids inhibit insulin activation of insulin receptor substrate-1 (IRS-1)-associated phosphatidylinositol 3-kinase activity in muscle. J Biol Chem 277: 50230-50236.*

8. *Holland WL, Brozinick JT, Wang LP, Hawkins ED, Sargent KM, et al. (2007) Inhibition of ceramide synthesis ameliorates glucocorticoid-, saturated-fat-, and obesity-induced insulin resistance. Cell Metab 5: 167-179.*
