# Supplementary figures and images for: Metabolic Network Topology Reveals Transcriptional Regulatory Signatures of Type 2 Diabetes
Source: PLoS Comput Biol. 2010 Apr 1;6(4):e1000729. doi: 10.1371/journal.pcbi.1000729 (PMC2848542; doi:10.1371/journal.pcbi.1000729)

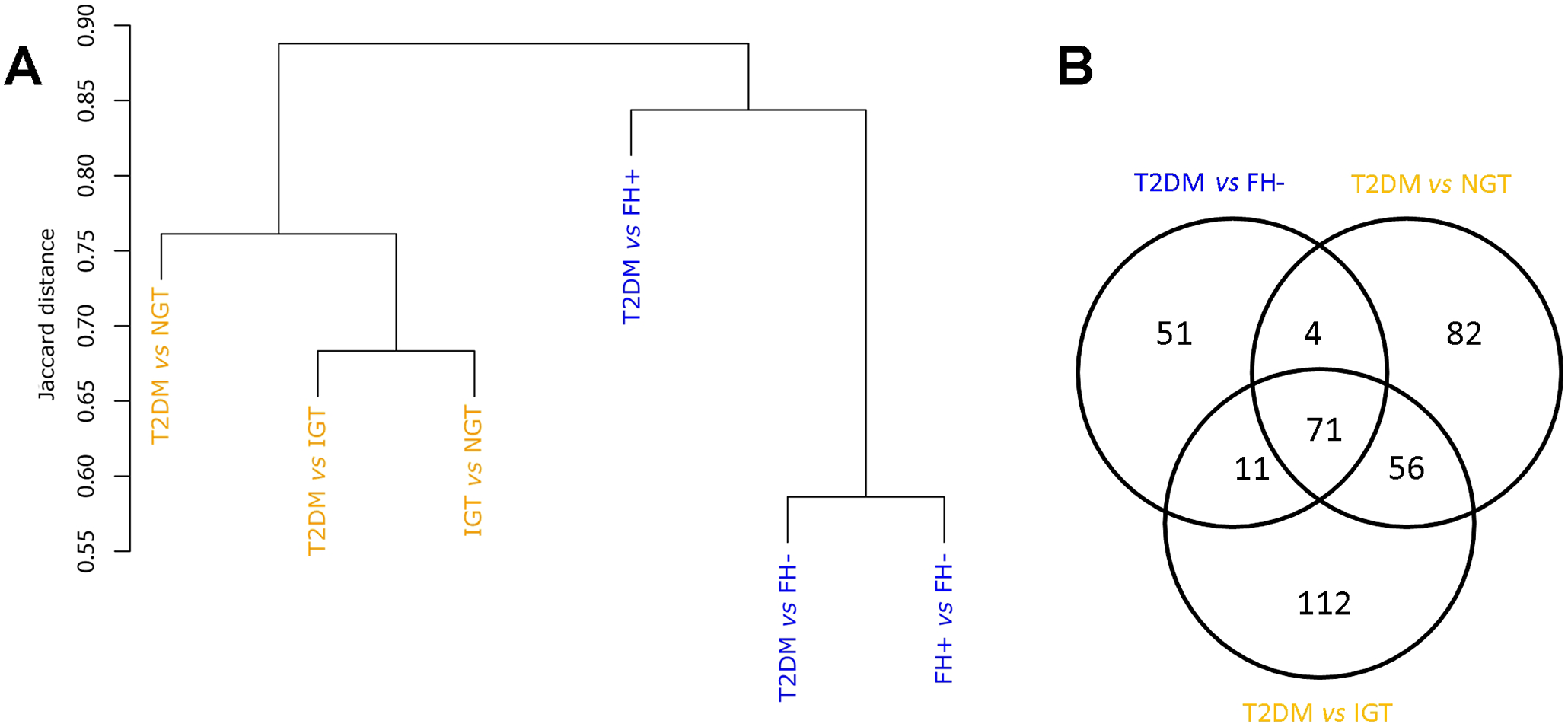

Supplement: Figure S1 — Hierarchical clustering of pair-wise comparisons within the Swedish male and Mexican-American datasets based on the overlapping reporter metabolites (EHMN network). (0.21 MB TIF) [file pcbi.1000729.s010.tif]
